# Post-Partum Hæmorrhage Treated by Injection of Hot Water

**Published:** 1878-06

**Authors:** 


Post-Partum Hæmorrhage Treated by Injection of Hot Water.—Dr. Atthill gives in the *London Lancet*, May 1878, the details of sixteen cases of Post-Partum Hæmorrhages treated by injection of hot water into the cavity of the uterus, and adds,—1st, that we do not, as a rule, employ hot water in cases of post-partum hæmorrhage till the application of cold has failed to arrest it; 2nd, that it is most markedly beneficial in the case of weakly, delicate women, and in those in whom, profuse hæmorrhage having been checked, blood continues to be lost in small quantities, the the uterus alternately relaxing and contracting; 3rd, that, for its successful application, the tube of the syringe must be carried fairly into the uterus; 4th, that the temperature of the water must not be under 110°.

In no single instance did any unpleasant symptom follow; and all the patients, with the exception of one case, stated that they experienced feelings of the greatest comfort and relief from the treatment. In the case referred to, in which pain followed the injection of the hot water, we were uncertain whether the ovum had been expelled or not. I had but once previously injected hot water in a case of abortion, but this woman was in a very critical state; she was cold and faint, and I stated to the class that if the ovum had not come away the hot water might stimulate the uterus to expel it, and that if the uterus were empty I believed it would arrest the draining which still continued. This case is, in my opinion, a very important one, and will lead me for the future to treat hæmorrhage occurring in cases of abortion in a similar manner.

